# Supplementary material for: Content-rich biological network constructed by mining PubMed abstracts
Source: BMC Bioinformatics. 2004 Oct 8;5:147. doi: 10.1186/1471-2105-5-147 (PMC528731; doi:10.1186/1471-2105-5-147)
Supplement: Additional File 2 — The original results of the above study (non-essential files are deleted to keep the file size under the limit set by BMC bioinformatics). [file 1471-2105-5-147-S2.bz2 › chilibotAdditionalFile2/dip50/61ID9187653E220/html/ELA1_CSN2.html]

 


 **ELA1** and **CSN2** 
  
Found 8 abstracts in PubMed, retrieved 8.  
 

 What does Google say? 
 PDF only 
| .edu only 

---

**Interactive relationship** (e.g. stimulation, inhibition, etc)

### Neutral relationship

- The in vitro kinetic studies of caseinate hydrolysis by elastase  [ **ELA1** ] , one of the main polymorphonuclear neutrophil PMN proteases, suggested that the beta casein  [ **CSN2** ]  peptide might be generated by elastase  [ **ELA1** ] .  Ref: 12741540 J Dairy Sci, 2003
- Mass spectrometric screening reveals that an unmodified natural heptapeptide human beta casomorphin 7, an internal sequence of human beta casein  [ **CSN2** ]  that possesses opioid like activity reacts with porcine pancreatic elastase  [ **ELA1** ]  to form an unusually stable acyl enzyme complex at low pH.  Ref: 9187653 Nat Struct Biol, 1997
**Non-interactive relationship** (e.g. studied together, co-existance, homology, etc.)

- Kinetic and crystallographic analysis of complexes formed between elastase  [ **ELA1** ]  and peptides from beta casein  [ **CSN2** ] .  Ref: 11358514 Eur J Biochem, 2001
